# Production of oyster mushroom (*Pleurotus ostreatus*) from some waste lignocellulosic materials and FTIR characterization of structural changes

**DOI:** 10.1038/s41598-023-40200-x

**Published:** 2023-08-09

**Authors:** Caglar Akcay, Faik Ceylan, Recai Arslan

**Affiliations:** 1 Department of Forestry, Forestry Vocational School, Duzce University, Konuralp Campus, Duzce, Türkiye; 2 Industrial Recycling of Agricultural Wastes Application and Research Center, Duzce University, Konuralp Campus, Duzce, Türkiye

**Keywords:** Plant breeding, Chemical engineering, Biomaterials - proteins, Fungal biology, Fungal ecology, Fungal evolution

## Abstract

In this study, oyster (*Pleurotus ostreatus*) mushroom was cultivated from hazelnut branches (HB) (*Corylus avellana* L.), hazelnut husk (HH), wheat straw (WS), rice husk (RH) and spent coffee grounds (CG). Hazelnut branch waste was used for the first time in oyster mushroom cultivation. In the study, mushrooms were grown by preparing composts from 100 to 50% mixtures of each waste type. Yield, biological activity, spawn run time, total harvesting time and mushroom quality characteristics were determined from harvested mushroom caps. In addition, chemical analysis of lignocellulosic materials (extractive contents, holocellulose, α-cellulose, lignin and ash contents) were carried out as a result of mushroom production and their changes according to their initial amounts were examined. In addition, the changes in the structure of waste lignocellulosic materials were characterized by FTIR analysis. As a result of the study, 172 g/kg yield was found in wheat straw used as a control sample, while it was found as 255 g/kg in hazelnut branch pruning waste. The highest spawn run time (45 days) was determined in the compost prepared from the mixture of hazelnut husk and spent coffee ground wastes. This study showed that HB wastes can be used for the cultivation of oyster mushroom (*P. ostreatus*). After mushroom cultivation processes, holocelulose and α-cellulose content rates decreased while ash contents increased. FTIR spectroscopy indicated that significant changes occurred in the wavelengths regarding cellulose, hemicellulose and lignin components. Most significant changes occurred in 1735, 1625, 1510, 1322 and 1230 wavelengths.

## Introduction

There are about 2000 edible mushroom species worldwide. Some of these mushroom species can be grown every day of the year when suitable conditions are created. The most commonly cultivated mushroom species are white button mushroom (*Agaricus bisporus*), oyster mushroom (*Pleurotus* spp.) and shiitake mushroom (*Lentinula edodes*)^1^. Oyster mushrooms reduce the blood glucose level and reduce the risk of cancer with its high level of nutrients^1,2^. Oyster mushrooms are the easiest to cultivate and have the shortest growing period when the necessary conditions are met. In addition, the costs required for cultivation are financially lower than other mushroom species. Because it can be easily grown on organic agricultural waste-based substrates. Compared to other mushroom species, it can be grown on very different substrate materials^1,3,4^. Normally, substrates rich in lignocellulosic materials such as straw, sawdust and cotton waste are preferred for growing mushrooms^3,5,6^. However, as substrates, palm cones, corn cobs^7^, sugarcane pulp, coconut fiber, sugarcane pulp and their combinations^3^, chickpea straw and sunflower heads^8^, cardboard and plant fiber^9^, hazelnut leaves, tilia leaves, wheat straw and European poplar leaves^5^ have also been tested and used in oyster mushroom cultivation so far. Among these substrates, wheat straw material is rich in lignin, cellulose and hemicellulose and can provide nutrients for mycelial growth and fruit formation. It has also been stated that it provides high biological efficiency^5^. However, according to Girmay^9^ and Mandeel et al.^10^, it was stated that sawdust exhibits low yield and performance, and the reason for this is that sawdust with low protein content is insufficient for fungal growth.

Although many lignocellulosic materials have the potential to cultivate oyster mushrooms, the nutrient content of lignocellulosic material appears to be a factor that significantly affects mushroom yield and growth. Substrates containing high lignin and cellulose have been reported to prolong the mushroom harvesting time, while those with high nutrient content have been reported to facilitate the colonization process of fungi compared to those with low content. It is stated that substrates with low nutrient content will cause contamination such as green mold^11^. Although all kinds of lignocellulosic materials can be used in oyster mushroom cultivation, some lignocellulosic materials may differ between countries and even regions in terms of availability^5,12^.

Turkey and Italy constitute the majority (80%) of the world hazelnut (*Corylus avellana* L.) production^13,14^. In 2019, 1.125.178 tons of hazelnuts were produced worldwide^14^. Hazelnut husk and hazelnut branch pruning wastes appear after hazelnut harvest. Hazelnut husk and hazelnut branch are renewable resources and are not used in any field in the forest industry. For this reason, sufficient studies have not been done in the literature studies. It is used by local farmers by burning it in houses for heating purposes or it is used as a soil conditioner after harvest. However, burning for heating purposes causes environmental problems because it causes air pollution. As a result of hazelnut production, 1/5 of 1 kg of dried hazelnut comes out as husk. It is reported that approximately 3 × 10^5^ tons of hazelnut shells (and husk) are released annually in Turkey^15–17^. Hazelnut husk and branch wastes are lignocellulosic materials and have a fibrous structure containing cellulose, hemicellulose and lignin^17^. This by-product (waste) can be used as a substrate for the cultivation of lignocellulosic fungi due to its high lignin content^14^.

While hazelnut husk waste has recently been used in mushroom cultivation, there is no reference to the use of hazelnut pruning wastes in mushroom cultivation. Hazelnut pruning wastes are usually caused by cutting old branches and newly born branches^18^. According to 2003 year data, it was reported that as a result of 0.65 million tons of hazelnut production, 0.45 million tons of hazelnut hard shell and 1.7 million tons of pruning waste were released^19,20^.

Coffee grounds waste is also a substrate that has recently been used for mushroom cultivation. It has been reported that 6 million tons of coffee grounds waste occur annually in the world. Cultivation of *P.ostreatus* from coffee grounds waste is seen as a new method for recycling this waste in developed countries^21^. Since coffee grounds contains 12.40% cellulose, 39.10% hemicellulose and 23.90% lignin, rot fungi can develop on its grounds^22^.

The purpose of this study is to cultivate medicinal and edible oyster mushroom (*P. ostreatus*) from some waste materials such as hazelnut branch pruning wastes, hazelnut husk, wheat straw, coffee grounds and rice husks. In the study, hazelnut branch pruning wastes were used for the first time in mushroom cultivation. In addition to the quality analyzes of the grown mushrooms, chemical changes (holocellulose, alpha cellulose, lignin amounts extractive substances and ash contents) in lignocellulosic materials were determined before and after mushroom cultivation. Chemical changes in lignocellulosic materials due to *P. ostreatus* activity were also characterized by FTIR spectrometry.

## Materials and methods

### Preparation of composts from lignocellulosic materials

Hazelnut branch/pruning wastes (*Corylus avellana* L.), hazelnut husk, wheat straw, spent coffee ground and rice husk wastes were used to cultivate Oyster mushrooms in the study. Hazelnut brunch (HB), hazelnut husk (HH) and wheat straw (WS) were supplied from growers and spent coffee ground (CG) was supplied from a local coffee company in the Düzce region in Türkiye. HB and HH were grinded in Wiley mill by the size of 1 cm. WS was used by size 5–6 cm and CG was 0.3–0.5 mm.

Air-dry 100% and 50% by weight homogeneous mixtures were prepared (w/w) (Table 1). The mixtures were wetted at regular intervals for 1 day, and their moisture was ensured to reach 50–55%. Then, 1 kg of each combination is weighed and filled into heat resistant polypropylene bags (28 × 42 cm) and kept in an autoclave at 121 °C for 1.5 h. 5 replicates were autoclaved for each combination. After sterilization, the pH and moisture contents of each mixture were determined. The autoclaved composts were left to cool overnight and allowed to reach a temperature of 24 °C (room temperature). Then, *P. ostreatus* spawn was inoculated into the composts at a rate of 2% compared to the dry compost weight in the air chamber. *P. ostreatus* spawn used in the study were purchased from Bursa Mantar company, Bursa, Türkiye. The spawn inoculated composts were mixed homogeneously and the bags were tightly tied and transferred to incubation room.

**Table 1:** Substrates used in the study and pH values.

Substrates	Symbol	pH
Hazelnut branch	HB	7.10
Hazelnut husk	HH	6.04
Wheat straw	WS	7.40
Coffee ground	CG	6.64
Rice husk	RH	8.00
Hazelnut branch + Hazelnut husk (1:1)	1HB:1HH	6.67
Hazelnut branch + Wheat straw (1:1)	1HB:1WS	7.00
Hazelnut branch + Coffee ground (1:1)	1HB:1CG	6.94
Hazelnut branch + Rice husk (1:1)	1HB:1RH	7.37
Hazelnut husk + Coffee ground (1:1)	1HH:1CG	5.97
Wheat straw + Hazelnut husk (1:1)	1WS:1HH	6.57
Wheat straw + Coffee ground (1:1)	1WS:1CG	7.80
Rice husk + Coffee ground (1:1)	1RH:1CG	6.82

### Incubation and harvest

The composts were kept in a dark environment at 22 ± 2 °C and 70% relative humidity in the air-conditioning room. After the mycelium colonization was completed, holes were drilled on the bags and the room temperature was set to 14–16 °C. Room humidity is increased to 80–90%. After this stage, incubation room was ventilated (with the CO_2_ level below 1000 ppm) and after the promordium formation was observed, 50–60 lx light per m^2^ was given to the room in order to promote the mushroom cap formation. The mushroom caps were harvested and were weighed on precision scales and their wet weights were recorded. A total of 3 flush mushrooms were harvested from each combination. Yield and biological activities of each combination were determined according to the mushroom cap weights with the following formula^23^.

1 $$\left( {{\text{Weight}}\;{\text{of}}\;{\text{fresh}}\;{\text{mushrooms}}\;{\text{harvested}}\;{\text{gr/}}1\;{\text{kg}}\;{\text{substrate}}} \right)$$

2 $$\left( {{\text{Weight}}\;{\text{of}}\;{\text{fresh}}\;{\text{mushrooms}}\;{\text{harvested}}/{\text{dry}}\;{\text{matter}}\;{\text{content}}\;{\text{of}}\;{\text{the}}\;{\text{substrate}}} \right) \times {1}00$$

In addition, spawn run time, days to first harvest (earliness) and total harvest time were recorded.

### Nutrient content analysis of mushrooms

Ash, dry matter, moisture, oil, nitrogen, protein and element analysis of the harvested mushrooms were carried out in Scientific and Technological Research Application and Research Center (DUBIT) laboratory, Duzce, Turkey. Ash, dry matter, moisture, oil, nitrogen, protein and element analysis of the harvested mushrooms were determined according to Kacar^24^.

### Chemical analysis of lignocellulosic materials

#### Determination of extractives

The raw control materials used before mushroom production (HB-C, HH-C, WS-C, CG-C and RH-C) and the remaining fungal degraded materials (HB-F, HH-F, WS-F, CG-F and RH-F) were ground and sieved in the Wiley mill. 40 mesh samples were dried in an oven (103 °C ± 2) and prepared for extractive content determination. 5 g of full dry samples were weighed and subjected to the acetone solvent extraction process for 6 h. Three replicates of each substrate type were carried out. After the extraction process was completed, it was vacuum filtered from the crucible (pore 2) and dried at 103 °C ± 2 for 12 h. The amount of extractive substance in the lignocellulosic materials was determined compared to the initial full dry weight. Extractive content of the substrates was determined according to TAPPI T 204 cm-17^25^ with some modifications.

#### Determination holocellulose content

Holocellulose determination was carried out according to the chloride method of Wise and John^26^. The method was applied to 5 different raw materials; hazelnut pruning waste, hazelnut husk, rice husk, coffee grounds and weat straw materials. Oven-dried extractive free 40-mesh samples (5 g) were placed in a 250 mL flask containing 160 mL of distilled water, 1.5 g of NaClO_2_, and 0.5 mL of glacial acetic acid and incubated at 78 °C for a period of time. The flask was shaken for 1 h and stirred at regular intervals throughout the reaction. After 1 h, 1.5 g of NaClO_2_ and 0.5 mL of glacial acetic acid were added to the mixture and heating was continued for 1 h. This process was repeated four times and when chlorination was complete, the mixture was filtered through a glass crucible (por 2). After the residue was washed repeatedly with acetone followed by cold distilled water, then dried in an oven at 103 ± 2 °C. The holocellulose content (%) was then determined relative to the initial full dry weight.

#### Determination alpha-cellulose content

Alpha-cellulose content was determined according to TAPPI T 203 cm-09^27^ standard using 17.5% NaOH on holocellulose samples. About 2 g of oven-dried holocellulose were placed in a beaker and 10 mL of 17.5% NaOH solution was added. This mixture was mixed twice with 5 mL of 17.5% NaOH solution at 5-min intervals and then kept in a water bath at 20 °C for 30 min. Then, 33 mL of distilled water was added to the mixture and kept at 20 °C for 60 min, and then filtered through a por 2 crucible. The residue in the crucible was first washed with 100 mL of 8.3% NaOH solution, then with 15 mL of 10% acetic acid and 250 mL of distilled water, and dried at 105 ± 3 °C and weighed. Finally, the % a-cellulose content was determined relative to oven dried holocellulose.

#### Determination of lignin content

The amount of removed component from the wood by chloritization from the extractive-free material in holocellulose determination was accepted as lignin. This is the theoretical lignin content.

#### Fourier-transform infrared spectroscopy (FTIR) assesment

FTIR (Fourier-transform infrared spectroscopy) is a fast and non-destructive technique that has been successfully used to detect chemical compounds in complex structures. The most intriguing application of this technique is currently seen as explaining the degradation processes of various agricultural wastes for mushroom cultivation^14^.

FT-IR analyzes were performed in Düzce University Scientific and Technological Research Laboratory. Hazelnut branch, hazelnut husk, wheat straw, coffee grounds and rice husks were ground before analysis and dried at 103 °C ± 2 for 12 h. The absorption spectra of the substrates used were obtained by the KBr (Potassium Bromide) technique based on pellet formation. Since this method gives less noisy peaks than the methods obtained with other ATR, FT-IR (4 cm^−1^, 40 scan) was preferred. In each analysis, approximately 5–10 mg of substrate and 1% of the substrate weight of KBr powder were mixed and pressed into pellets for the FT-IR measurement.

### Statical analysis

Differences in yield, biological efficency, spawn run time, and total harvest time of the mushrooms obtained according to the type of waste lignocellulosic material and the differences between the chemical components of the lignocellulosics were evaluated with one-way ANOVA. Duncan's means discrimination test was applied at the level of α = 0.05 for the variables determined to have differences between the groups according to the ANOVA results.

### Research involving plants

The authors complied with the IUCN Policy Statement on Research involving Endangered Species and the Convention on Trade in Endangered Species of Wild Fauna and Flora.

## Results and discussion

Table 2 shows the yield and biological efficiency values for the each substrate. According to the Table 2, the differences between the substrates in terms of mushroom yield and biological activity values were found to be statistically significant (*P* < 0.05). The highest yield (257 g/kg) and biological efficiency (64%) values were determined in mixtures of 1RH: 1CG. In addition, 255.7 g/kg yield value and 63.9% biological efficiency were determined in the substrate prepared from HB alone. There was no statistical difference between the yield and biological efficiency values of mushrooms produced from HB alone and that of 1RH: 1CG mixtures (*P* < 0.005). The lowest yield was obtained in the substrates prepared by mixing HB and CG. In the literature studies, wheat straw is used as a control sample in the cultivation of *P. ostreatus*. In this study, a yield of 172.5 g/kg was found in WS alone. When HB and CG were added to the WS substrates, the yield and biological efficiency value increased, while decreased with the addition of HH substrates.

**Table 2:** Yield and BE values of substrates prepared with different mixture ratio.

Substrate	Yield (g/kg)	Biological efficiency/BE (%)
HB	255.7 *a* (22.5)	63.9 *a* (5.6)
HH	157.5 *bc* (46.5)	39.4 *bc* (11.6)
WS	172.5 *abc* (8.2)	43.1 *abc* (2.0)
CG	174.4 *abc* (20.1)	43.6 *abc* (5.0)
RH	182.6 *abc* (12.7)	45.6 *abc* (3.1)
HB:HH	166.2 *abc* (8.6)	41.6 *abc* (2.1)
HB:WS	208.7 *ab* (13.6)	52.2 *ab* (3.3)
HB:CG	186.6 *abc* (26.8)	46.6 *abc* (6.7)
HB:RH	153.0 *ab* (23.9)	38.3 *bc* (5.9)
HH:CG	105.2 *c* (32.3)	26.3 *cd* (8.0)
WS:HH	155.3 *bc* (24.6)	11.3 *d* (1.5)
WS:CG	236.9 *ab* (26.1)	59.2 *ab* (6.5)
RH:CG	257.0 *a* (79)	64.3 *a* (19.7)

Different letters in the same column indicate that there is no statistical difference between them. Values in parentheses indicate standard error.

When WS, RH, CG and HH were added to the HB substrates, the yield and biological efficiency value of these mixtures decreased. While the yield value of mushrooms prepared with CG alone was higher than those produced with 1:1 ratio ones, the yield and biological efficiency value of the combinations produced only with RH and CG were lower than CG alone.

It has been revealed by many studies that yield and biological efficiency values differ according to the type of substrate used. In literature studies, wheat straw is used as a control sample in mushroom cultivation. The mushroom yield obtained from the HB used for the first time in this study was found to be higher than that of WS, but there was no statistical difference between them. When WS and HB are used as a mixture in a ratio of 1:1, there was no statistical difference between them in terms of yield and biological efficiency. For this reason, it can be recommended to use HB as an alternative to WS or using their combinations. Pekşen and Küçükomuzlu^28^ revealed that mixtures prepared with hazelnut husk gave lower biological activity than wheat straw. Yildiz et al.^5^ obtained the highest yield and biological efficiency values in mixtures of wheat straw and waste paper prepared with hazelnut leaves, sawdust, wheat straw, waste paper, European poplar leaves and tilia leaves substrates. Fanadzo et al.^29^ studied wheat straw (*Triticum aestivum*), maize stover (*Zea mays* L.), thatch grass (*Hyparrhenia filipendula*) and oil/protein rich supplements ((maize bran, cottonseed hul (*Gossypium hirsutum*)) and they stated that maize stover is a more suitable lignocellulosic material for the cultivation of *P. ostreatus* than wheat straw.

Table 3 shows spawn runtime, days to first harvest time (earliness) and total harvest time according to the substrate types used in the study. Statistically significant differences were found between spawn run times, earliness and total harvest time of the substrates (*P* < 0.005). According to Table 3, the longest spawn run time (45 days) was determined in substrates prepared with 1HH:1CG while the lowest spawn run time was 15 days in the substrates prepared with 1RH:1CG. Spawn run times did not differ statistically between mixtures of HB, WS, 1HB:1WS, 1HB:1CG, 1HB:1RH and 1RH:1CG. The first harvest times (earliness) were also similar to the spawn run time. When the average total harvest times are examined, the highest harvesting time (83 days) was obtained in the substrates prepared from the mixture of 1HB:1HB, while the lowest (41.7 days) determined in the mixtures prepared with HB and WS.

**Table 3:** Spawn run time, earliness and total harvest times according to substrate types.

Substrates	Mean spawn run time (day)	Mean earliness (day)	Mean total harvest time (day)
HB	19.8 *ab*	26.7 *ab*	69.3 *bc*
HH	27.0 *cd*	34.0 *cd*	76.5 *cde*
WS	20.0 *ab*	27.0 *ab*	67.0 *bc*
CG	32.7 *de*	39.7 *de*	62.7 *b*
RH	26.7 *cd*	33.7 *cd*	62.3 *b*
HB:HH	32.7 *de*	39.7 *de*	83.1 *e*
HB:WS	17.3 *a*	24.3 *a*	41.7 *a*
HB:CG	15.7 *a*	22.7 *a*	45.0 *a*
HB:RH	17.0 *a*	24.0 *a*	68.0 *bc*
HH:CG	45.0 *f*	52.0 *f*	82.3 *de*
WS:HH	25.0 *bc*	32.0 *bc*	72.8 *cd*
WS:CG	37.0 *e*	44.0 *e*	81.0 *de*
RS:CG	15.0 *a*	22.0* a*	48.3 *a*

When lignocellulosic material was used alone, the lowest spawn run time was found in WS and HB substrates. The short spawn run time in wheat straw is due to the shorter fermentation time and less nutrient requirement since it contains 39/51% cellulose, 76% holocellulose, 18% lignin, 3.5% protein and 0.6% digestible protein^5^. The long spawn run time in substrates alone and mixtures of CG and HH may cause the development of green molds due to contamination and a decrease in yield values^30^. High spawn run time in HH was revealed by Puliga et al.^14^ as well. On the other hand, Carrasco-Cabrera et al.^21^ reported that coffee grounds increase spawn run time. The long spawn run time in coffee wastes may be due to the very thin and small size of the coffee particle geometry^31^.

The properties of some nutrients (ash, dry matter, moisture, oil, nitrogen and protein) of mushrooms obtained from substrates are shown in Table 4. When nutrient values of mushrooms obtained from HB were examined, ash and dry matter were generally higher than that of other substrates, while moisture value was lower. When protein and nitrogen contents are considered, the amount of nitrogen and protein in mushrooms obtained from coffee to coffee supplemented substrates was generally found to be high. The lowest amount of protein and nitrogen was determined in mushrooms cultivated from wheat straw alone. In cases where protein needs are required, mushrooms can be produced by supplementing with CGs. The highest amount of oil was determined from mushrooms produced from CG alone, while the lowest was obtained in mixtures of 1HB:1RH.

**Table 4:** Some chemical properties of the mushrooms obtained substrates prepared by alone and different mixtures.

Substrates	%					
	Ash	Dry matter	Moisture	Oil	N	Protein
HB	7.465	20	80	2.866	5.09	22.3
HH	6.576	17.1	82.9	2.64	4.08	17.9
WS	6.728	13	87	2.557	3.9	17.1
CG	5.743	18.44	81.56	3.845	5.9	25.8
RH	4.809	13	87	3.036	4.54	19.9
HB:HH	6.842	16.71	83.29	3.109	4.57	20
HB:WS	5.432	9.44	90.56	2.344	3.9	17.1
HB:CG	5.571	12.11	87.89	2.224	5.45	23.9
HB:RH	6.15	20.95	79.05	1.993	5.18	22.7
RH:CG	5.329	13.2	86.8	3.089	5.89	25.8
WS:HH	8.001	17.43	82.57	3.429	5.15	22.6
WS:CG	6.269	12.66	87.34	3.038	7.63	33.4

Elemental contents of mushrooms obtained from substrates prepared from different types of lignocellulosic wastes are given in Table 5. Significant differences were detected between the elemental contents of the mushrooms obtained from the substrates. The highest amounts of the nutrients P (26,288 mg/kg), Mg (3510 mg/kg), K (48,347 mg/kg), Fe (133.89 mg/kg), Mn (15.85 mg/kg), Cu (33.29 mg/kg) and Zn (98.26 mg/kg) were detected in mushrooms cultivated from HH substrate. In the study, Na (746 mg/kg) element in mushrooms obtained from HB waste which was used in mushroom cultivation for the first time was found to be higher than mushrooms obtained from the other substrates. The amount of Mn in mushrooms obtained from HB was found to be 5.19 mg/kg, that lower than mushrooms obtained from other substrates. Mineral analysis for *P.ostreatus* in this study resembled those recorded by Ananbeh and Almomany^32^.

**Table 5:** Elemental analysis of harvested mushrooms.

Substrates	P	Mg	Na	Ca	K	Fe	Mn	Cu	Zn
	(mg/kg)	(mg/kg)	(mg/kg)	(mg/kg)	(mg/kg)	(mg/kg)	(mg/kg)	(mg/kg)	(mg/kg)
HB	7809	1340	746	50.72	29,245	79.34	5.19	10.83	37.03
HH	26,288	3510	678	63.95	48,347	133.89	15.85	33.29	98.26
WS	14,783	2250	652	63.48	37,852	98.99	8.45	16.94	64.44
CG	20,512	2315	581	45.00	30,833	86.88	11.72	28.71	73.61
RH	13,199	1966	595	74.44	31,307	63.83	6.91	4.73	44.01
1HB:1HH	12,408	1405	377	44.99	25,078	64.25	5.98	11.27	36.83
1HB:1WS	9763	1334	375	40.34	24,279	47.39	5.19	7.90	30.20
1HB:1CG	15,274	2094	444	34.43	29,161	55.82	9.58	11.60	53.96
1HB:1RH	10,629	1648	474	31.20	31,862	60.14	6.14	6.44	37.12
1HH:1CG	12,692	1770	621	69.52	29,500	47.74	6.71	7.86	36.92
1WS:1HH	13,741	1951	298	72.07	31,296	69.02	8.07	14.30	49.45
1WS:1CG	9107	2005	588	23.60	34,003	73.72	6.17	9.48	46.77

### Chemical analysis of lignocellulosic materials used in the study

**Figure 1:**
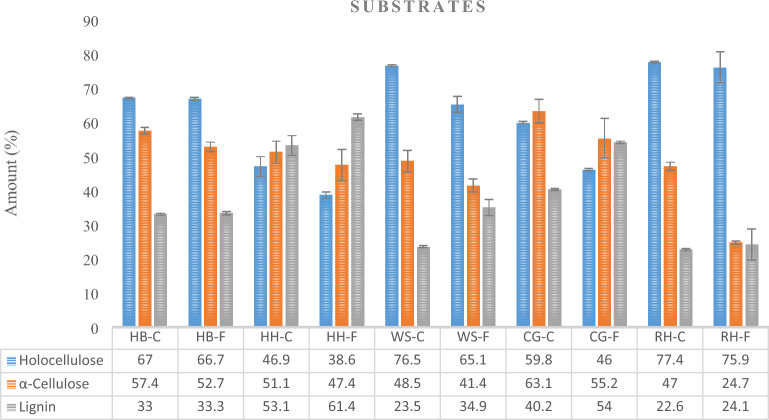
Chemical analysis findings of before and after mushroom cultivation according to substrate types. *Note* C, Control for each substrate/before cultivation; F, Fungal degraded for each substrate/after cultivation (HB-C: Hazelnut branch control /before mushroom cultivation; HB-F, Hazelnut branch fungal degradation/after mushroom cultivation (the other substrates were designed similarly in the Figure)).

According to the substrate types, the amounts of holocellulose, alpha cellulose and lignin before and after mushroom cultivation are shown in Fig. 1. Among the substrate types, the highest amount of holocellulose (77.4%) before fungal degradation was detected in the RH-C (Rice Husk Control) sample, while the lowest was detected by 46.9% in the HH-C (Hazelnut Husk Control). It was observed that the amount of holocellulose decreased after fungal degradation. In the study, 67% holocellulose, 57.4% alpha cellulose and 33% lignin were detected in HB material, which was evaluated for the first time in mushroom production. In a previous study cunducted by Gençer and Özgül^33^, holocellulose was found to be 82.07%, alpha cellulose 41.33, Lignin 15.89%, ash 0.72% and extractive substance 2.83% in the HB undegraded control sample. The amounts of holocellulose, alpha cellulose, lignin and ash in the HH undegraded control sample were determined as 55.1%, 34.5%, 35.1% and 8.22%, respectively, in a previous study cunducted by Güney^17^. When the alpha cellulose ratios of the substrates were examined, the highest alpha cellulose ratio was found in the CG substrate type among the control samples. A decrease in alpha cellulose was observed in all substrate types after fungal degradation. When the lignin amounts were examined, there was an increase in the lignin ratio after the fungal attack compared to the control samples with the effect of degradation.

The decrease in holocellulose and alpha cellulose ratios was also indicated in a study conducted by Atila^34^ and Jonathan et al.^35^. The decrease in macromolecules in lignocellulosic materials after mushroom production is due to the use of these structures by *P.ostreatus* both during mycelial growth and during fruitbody formation. *P. ostreatus* fungal hyphae secrete large amounts of extracellular enzymes that cause degradation of cellulose, hemicellulose and lignin^36^. However, since the secreted enzymes act selectively, they can degrade the cell wall components at different rates. The decrease in the amount of holocellulose in the HB substrate was lower than in the other substrates. This may be due to the fact that the HB substrate has a more dense and compact structure. It can be said that the reason for the proportional increase or constant rather than the decrease in the amount of lignin is that lignin is more difficult to decompose than cellulose and hemicellulose due to its complex structure^37^.

Lignin plays a critical role carbon cycling on earth. Its heterogeneous structure provides rigidity to plants and protects cellulose and hemicellulose from degradation^38,39^. This rigid structure effected the oyster mushroom yield and biological efficiency in the study. HB gave the highest yield and biological efficiency of 255,7 g, 63.9%, while HH gave the lowest of yield and biological efficiency of 157.5 g, 39.4%, respectively. When the lignocellulosic contents of the substrates with the yield of mushrooms produced were compared it was observed that HB had the high α-cellulose content of 57.4% and low lignin content of 30%. Addition, HH had the highest lignin content of 53.1% in the current study. This findings can be atrubuted that since lignin component of the substrates is a heterogeneous and irregular arrangement of phenylpropanol polymer, it resists enzymatic degradation of fungus and protects the cellulose component. When cellulose component is digested, glucose and cellobiose sugars are produced that allows to consume by fungi. It seems that consumption of the sugars by the fungi (*P. ostreatus*) was limited by the lignin^40^.

Extractive contents, ash, and pH values of the substrates used in the study are shown in Table 6. When extractive contents were examined, the highest extractve content (12.7%) were detected in CG-C among the undegraded substrates while lowest extractive (0.07%) was detected in RH-C. According to obtained data from the study, extractive contents significantly increased in the HB-F, WS-F and RH-F compared to their undegraded controls in contrast to HH-F and CG-F (*P* < 0.05). The findings in Table 6 showed that ash content significantly increased in fungal degraded substrates (*P* < 0.05). The highest ash content was recored by 35. 5% in RH-F. After mushroom cultivation ash ratios increased by more than 100% in fungal degraded substrates compared to initial substrates. Similar results were also found in a study cunducted by Zhang and Fadel^41^. pH values of the substrates decreased when compared to initial values after fungal cultivation.

**Table 6:** Extractives, ash, and pH values of the substrates used in the study.

Substrates	Extractives (%)	Ash (%)	pH
HB-C	0.84 (0.14) b*	2.43 (0.23) *a*	7.1
HB-F	1.35 (0.44) *c*	4.97 (0.06) *b*	5.42
HH-C	1.74 (0.38) *cd*	9.07 (0.06)* c*	6.04
HH-F	1.55 (0.20)* cd*	18.9 (0.23) *d*	6.02
WS-C	1.41 (0.15) *c*	5.98 (0.28) *e*	7.4
WS-F	1.96 (0.35) *d*	16.5 (0.69)* f*	5.44
CG-C	12.7 (0.16) *e*	1.45 (0.30) *g*	6.64
CG-F	1.69 (0.31) *cd*	6.60 (0.13) *h*	5.67
RH-C	0.07 (0.01) *a*	16.9 (0.25) *f*	8.00
RH-F	0.44 (0.25) *ab*	35.5 (0.13) *ı*	6.21

C, Control for each substrate/before cultivation; F, Fungal degraded for each substrate/after cultivation, the values are standard deviations in parentheses, *different letters in the same column indicate that there is no statistical difference between them.

## FTIR assessment

**Figure 2:**
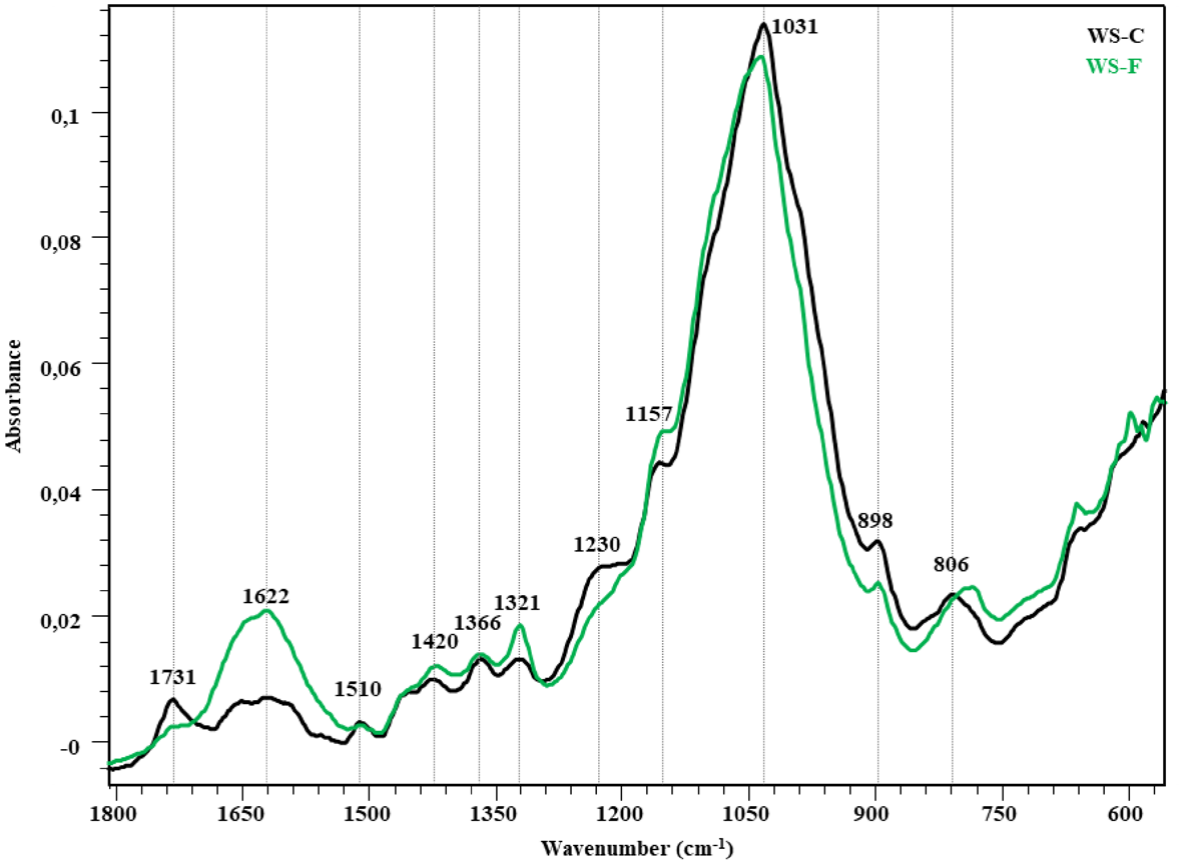
FTIR spectra of WS-C and WS-F (before and after fungal degradation).

**Figure 3:**
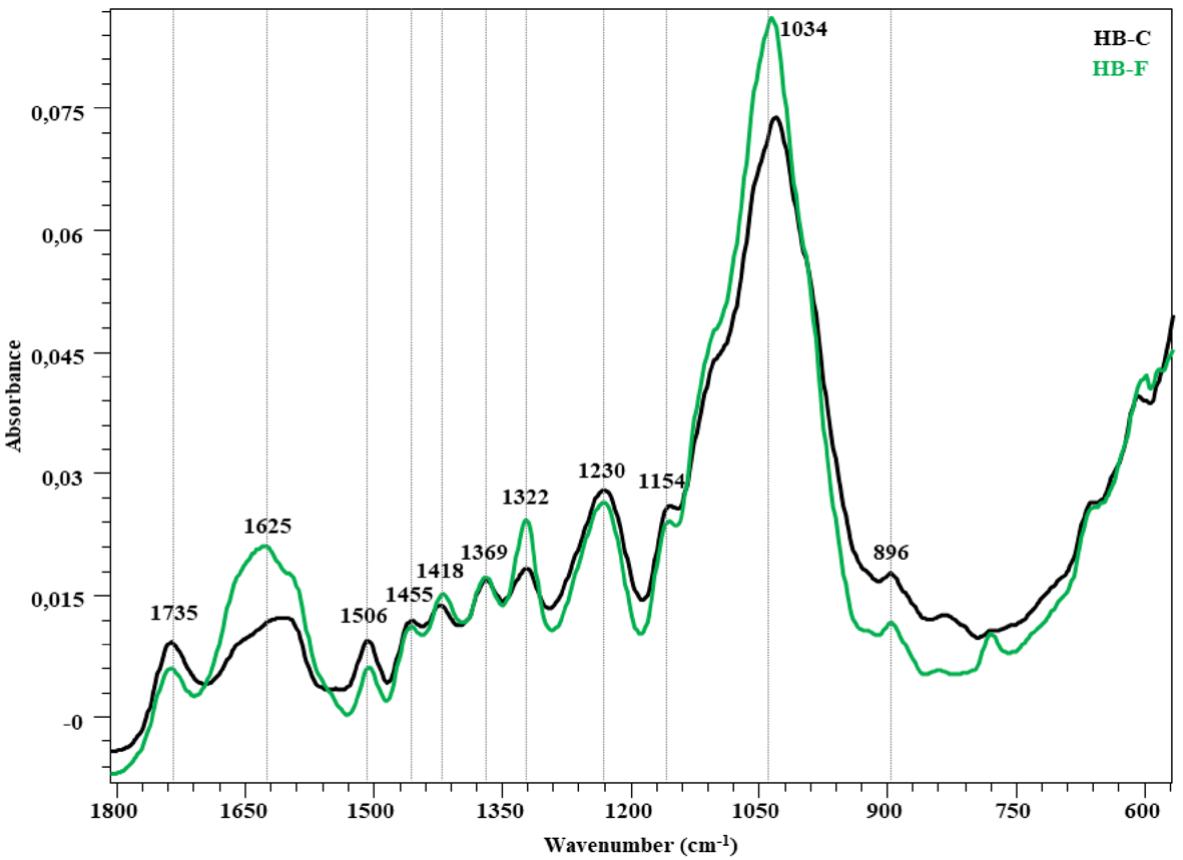
FTIR spectra of HB-C and HB-F (before and after fungal degradation).

**Figure 4:**
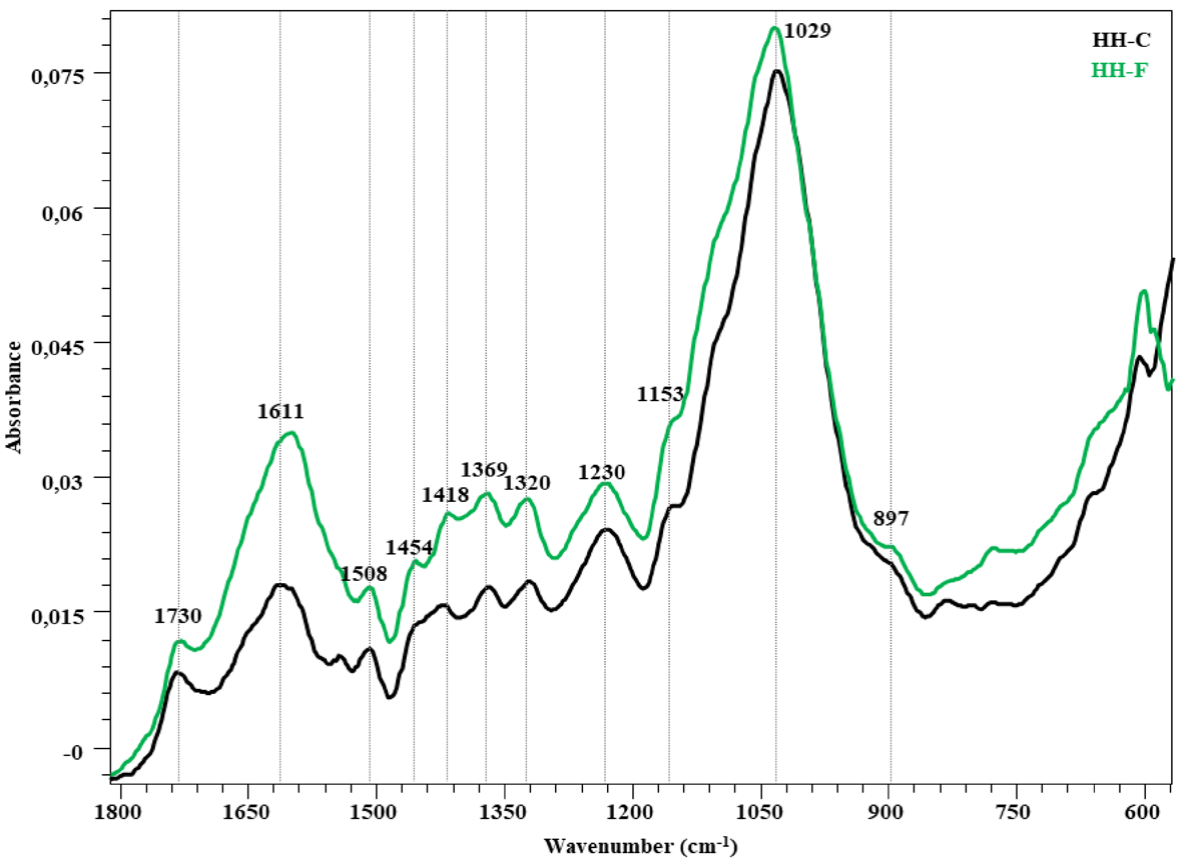
FTIR spectra of HH-C and HH-F (before and after fungal degradation).

**Figure 5:**
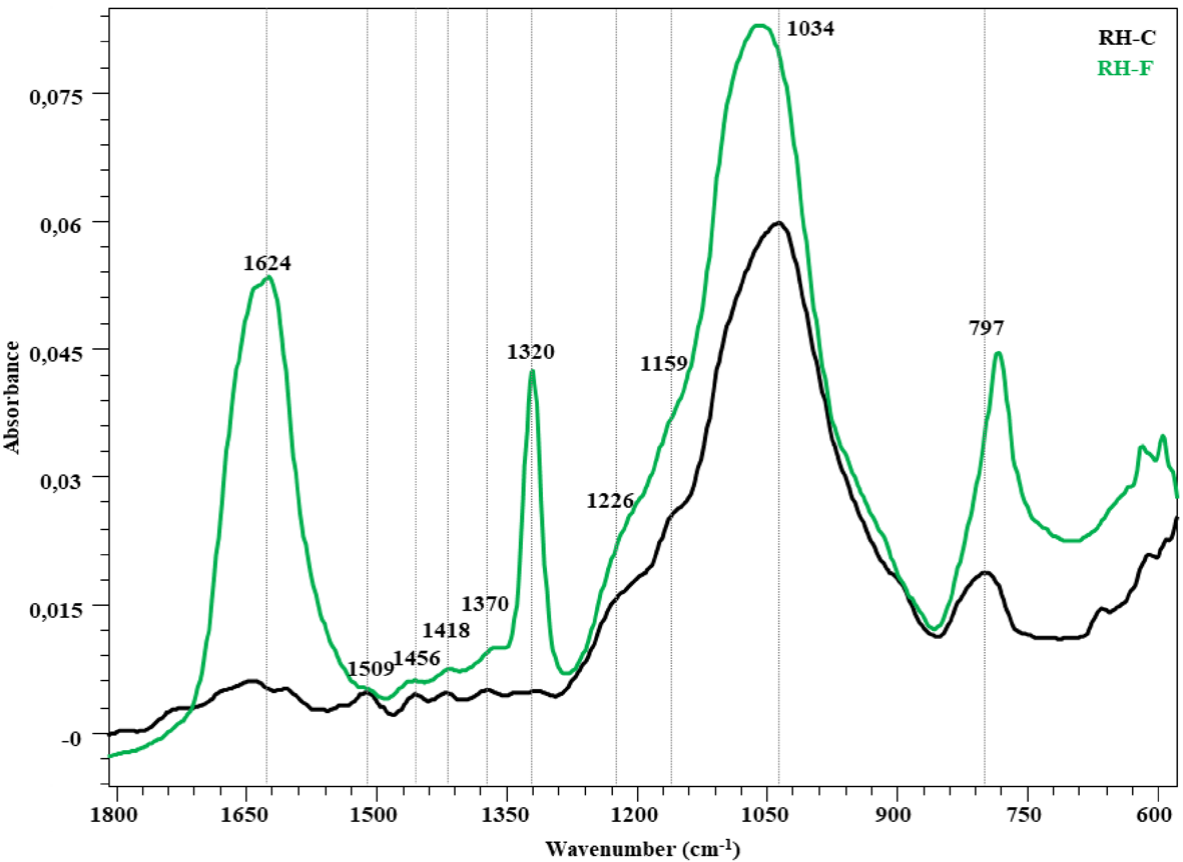
FTIR spectra of RH-C and RH-F (before and after fungal degradation).

The FTIR spectra in the fingerprint region (1800–600 cm^−1^) showing the changes in the structure after the fungal attack of the wheat straw, hazelnut branch, hazelnut husk and rice husk compared to the control are given in Figs. 2, 3, 4 and 5. The peaks occurring in this region represent lignin and polysaccharides in lignocellulosic materials (Table 7). The bands at 1730 cm^−1^ represent unconjugated C=O vibrations originating from the acetyl and carboxylic acid structures in xylan (hemicellulose)^46–48^. While a slight decrease was observed in the peak intensity of the wheat straw in this region, no significant change was observed in the other samples. Broadband in the range of 1680–1560 cm^−1^ represent the C=C and C=O stresses of the lignin aromatic chain (1630 cm^−1^)^48,52^, the C–O stretching of the lignin aromatic skeleton vibration (1595 cm^−1^)^46,47,53^ and the O–H deformation of the absorbed water (1640 cm^−1^)^46^. The overlap effect of these groups caused the formation of a broad peak. It is observed that this band intensity increased significantly in all samples. Visibility of vibrations in this region is increased by lignin degradation of *P. ostreatus*. The bands at 1510 cm^−1^ represent aromatic C–O stretching vibrations of lignin^46,50,53^. C–H deformations of CH_2_ and CH_3_ groups in lignin and hemicelluloses and CH_2_ in-plane bending vibrations of cellulose and lignin are observed in 1455 and 1418 cm^−1^ bands, respectively. The bands at 1370 cm^−1^ represent symmetrical and asymmetrical C–H deformation vibrations of cellulose and hemicelluloses^42,50,52,54^. The bands at 1320 cm^−1^ represent the CH_2_ in-plane bending vibration found at the C6 of the crystalline cellulose^50^. In all lignocellulosic materials, an increase in the intensity of these bands is observed with the fungal effect. This increase is related to the increase in peak lengths with crystalline regions appearing more prominently as *P. ostreatus* cellulose degrades the amorphous regions. It is seen that this effect is more in RH compared to other lignocellulosic materials. The band at 1230 cm^−1^ is associated with C–O stretching vibrations between lignin and xylan (hemicellulose)^47^. It represents the C–O–C stretching vibrations of shoulder glycosidic bonds at 1150 cm^−1^^56^, the C–O stretching of band cellulose and hemicelluloses at 1030 cm^−1^, and the C–OH bending vibration of xylan^52,54,57^. In the 1150–900 cm^−1^ band, there is no significant change for WS, HH and HB, while the band intensity increases significantly in RH. Significant changes in the 1624, 1320 and 1034 cm^−1^ wavelengths reveal the degradation effect of *P. ostreatus* on lignin, cellulose and hemicelluloses in RH. The weak shoulder at 898 cm^−1^ is associated with β-(1 → 4) glycosidic bonds of cellulose^48,52^.

**Table 7:** Evaluation of wavelengths in FTIR spectra of the substrates according to previous literature studies.

Wavenumber (cm^−1^)	Peak assignment	References
1743*	C=O stretching in aliphatic ester groups with associated lipids and quinic acids	^42–45^
1735	C=O stretching of unconjugated hemicellulose	^46–48^
1700–1600 region	C=C and C=O stretching in the hemicellulose (aromatic skeletal vibration)	^49,50^
1700–1650* region	C=C vibration of lipids and fatty acids	^42,45^
1650–1600* region	C=O stretching vibrations with associated caffeine	^45,51^
1523*	C=C vibration of aromatic rings from lignin	^42^
1640	absorbed water in the cellulose	^46^
1630	C=C and C=O stretching in the lignin aromatic ring	^48,52^
1595	C–O aromatic skeletal vibrations in lignin	^46,47,53^
1510	aromatic C–O stretching in lignin	^46,50,53^
1460–1400	CH_2_ and CH_3_ deformation in the lignin and hemicellulose, CH_2_ in-plane bending vibrations in the cellulose and lignin	^42,50,52,54^
1376, 1242, 1163, 1113, 1061*	chlorogenic acids (esters formed by quinic acid and certain trans-cinnamic acids)	^42^
1450–1150*	chlorogenic acids	^44,55^
1370	C-H symmetric and asymmetric deformation in cellulose	^47,50^
1320	C-H ring in-plane bending vibrations	^50^
1230	C-O stretching in lignin and xylan (hemicellulose)	^47^
1200–950 region	C–C and C–O stretching vibrations in pyranoid rings (characteristic for polysaccharides)	^42,56^
1160–1150	C–O–C stretching vibration of glycosidic bonds	^56^
1050–1030	C-O stretching (hemicellulose and cellulose), C–OH bending in xylan	^52,54,57^
950–700*	glycosidic bond β-(1 → 4) arabinogalactans, galactomannans, and cellulose	^58^
899	glycosidic bond β-(1 → 4) cellulose	^48,52^

*Belongs to only CG bands.

**Figure 6:**
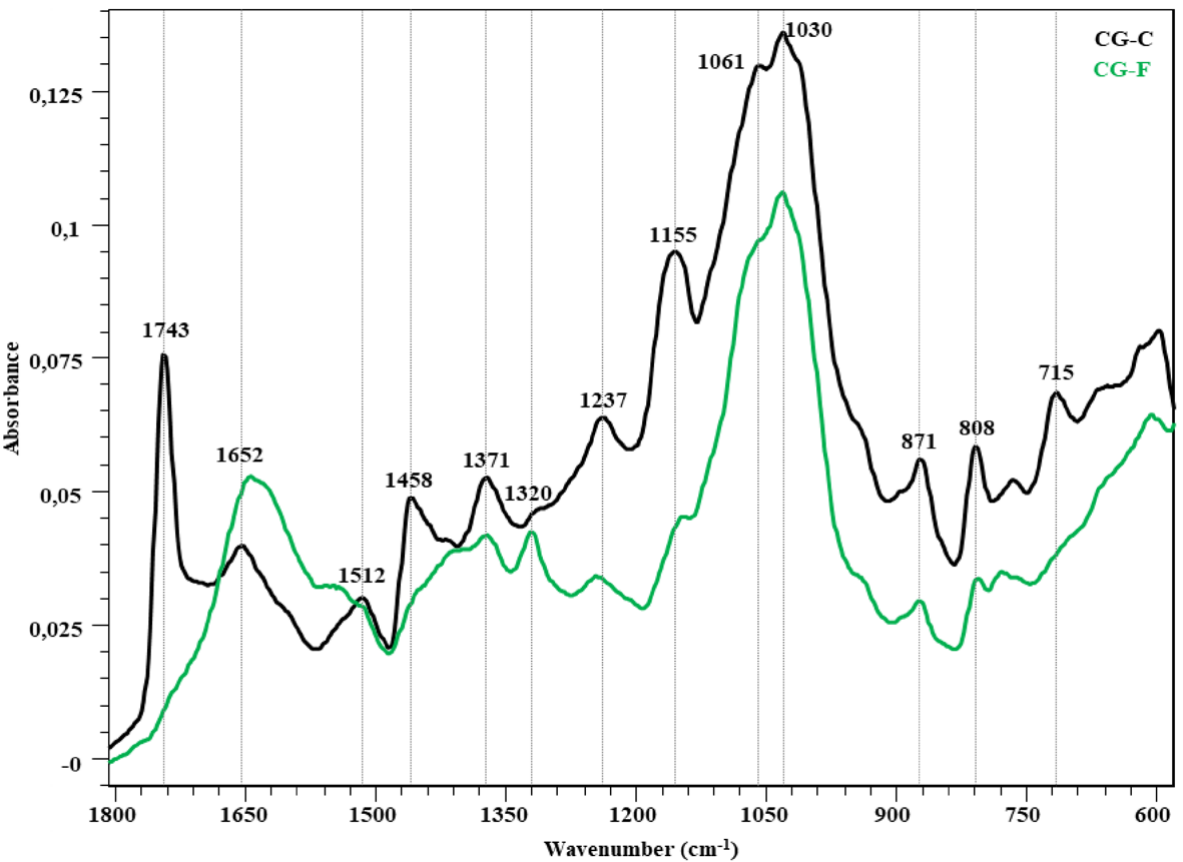
FTIR spectra of CG-C and CG-F (before and after fungal degradation).

FTIR spectra of CG-C and CG-F is shown in Fig. 6. The C=O stretching vibration of the CG-C (un-degraded control sample) is observed in the 1743 cm^−1^ band^42–45^. This band is characteristic of the aliphatic ester groups found in coffee-specific quinic acid and lipids. In this region (around 1735 cm^−1^ band), there is also a C=O stretching vibration of unconjugated hemicellulose. It is seen that this band disappears due to fungal attack (*P. ostreatus*). C=O stretching vibrations associated with caffeine and C=C vibrations of lipids and fatty acids are observed in the 1652 cm^−1^ band^42,45^. At the same time, this band represents the C=C and C=O stresses of the lignin aromatic chain (1630 cm^−1^), the C–O stretching of the lignin aromatic skeleton vibration (1595 cm^−1^) and the O–H deformation of the absorbed water (1640 cm^−1^)^45,51^. Visibility of vibrations in this region increased with lignin degradation. Aromatic C–O stretching vibration of lignin is observed in the 1512 cm^−1^ band^42^. In the 1458 cm^−1^ band, there are C–H bending and C–H deformation vibrations of the methyl and methylene groups found in chlorogenic acids together with lignin and polysaccharides. Bands of chlorogenic acids (esters formed by quinic acid and some trans-cinnamic acids) are observed in the 1450–1050 cm^−1^ region. The C–O deformation of quinic acid is observed in the 1061 cm^−1^ band, and the O–H deformation in the 1371 cm^−1^ band. In the 1237, 1155 and 1061 cm^−1^ bands, the absorption of the C–O–C ester bond of quinic acid takes place^44,55^. The three bands seen in the 900–750 cm^−1^ region represent β-(1 → 4) glycosidic bonds of arabinogalactans, galactomannans, and cellulose polysaccharides^58^. There is a decrease in the intensity of these bands. C–H, C–O–C, C–N and P–O vibration types specific to polysaccharides are also exhibited in the 900–1400 cm^−1^ region. Due to the overlap of bands specific to cellulose, hemicellulose and lignin, and chromatic acids, it is not possible to differentiate bands of these structures with FTIR. When considering the changes in the bands of the 1800–600 cm^−1^ fingerprint region, it is seen that *P. ostreatus* affects chlorogenic acids and cellulose, hemicellulose and lignin structures in lignocellulosic structure.

## Conclusion

In this study, *P. ostreatus* mushroom was cultivated for the first time from HB wastes. In addition, HH, CG, RH and WS were evaluated as substrate material by forming a mixture with HB wastes. As a result of the study, *P. ostreatus* mushroom was successfully grown in HB wastes. In addition, changes in the structure of lignocellulosic materials were characterized by chemical analyzes and FTIR studies. In the study, the highest yield value (255 g/kg) was determined in HB substrate, which was evaluated in mushroom cultivation for the first time while yield value was found to be 172 g/kg in WS used as control sample. α-cellulose and lignin content of the substrates effected yield and biological efficiencies of the mushrooms produced from the substrates alone. Spawn run time was found to be longer in HH and CG substrates compared to other substrates. After cultivation of *P. ostreatus* holocellulose and α-cellulose content rates decreased while lignin and ash content rates increased. FTIR spectroscopy assesments indicated that significant changes occured in the peaks of cellulose, hemicellulose and lignin components. Overall, this study showed that HB wastes can be used for the *P. ostreatus* cultivation.

## Data Availability

The datasets used and/or analysed during the current study available from the corresponding author on reasonable request.

## References

[1] Wan Mahari WA (2020). A review on valorization of oyster mushroom and waste generated in the mushroom cultivation industry. J. Hazard. Mater..

[2] Jedinak A, Sliva D (2008). *Pleurotus ostreatus* inhibits proliferation of human breast and colon cancer cells through p53-dependent as well as p53-independent pathway. Int. J. Oncol..

[3] Das D, Kadiruzzaman M, Adhikary S, Kabir M, Akhtaruzzaman M (2014). Yield performance of oyster mushroom (*Pleurotus ostreatus*) on different substrates. Bangladesh J. Agric. Res..

[4] Sanchez C (2010). Cultivation of *Pleurotus ostreatus* and other edible mushrooms. Appl. Microbiol. Biotechnol..

[5] Yildiz S, Yildiz ÜC, Gezer ED, Temiz A (2002). Some lignocellulosic wastes used as raw material in cultivation of the *Pleurotus ostreatus* culture mushroom. Process Biochem..

[6] Liaqat, R. *et al.* Growth and yield performance of oyster mushroom on different substrates. *Mycopath,* **12,** (2014)

[7] Samuel AA, Eugene TL (2012). Growth performance and yield of oyster mushroom (*Pleurotus Ostreatus* [sic]) on different substrate composition in Buea South West Cameroon. Sci. J. Biochem..

[8] Iqbal SH, Rauf CA, Sheikh MI (2005). Yield performance of oyster mushroom on different substrates. Int. J. Agric. Biol..

[9] Mandeel QA, Al-Laith AA, Mohamed SA (2005). Cultivation of oyster mushrooms (*Pleurotus* spp.) on various lignocellulosic wastes. World J. Microbiol. Biotechnol..

[10] Girmay Z, Gorems W, Birhanu G, Zewdie S (2016). Growth and yield performance of *Pleurotus ostreatus* (Jacq. Fr.) Kumm (oyster mushroom) on different substrates. AMB Express.

[11] Sofi B, Ahmad M, Khan M (2014). Effect of different grains and alternate substrates on oyster mushroom (*Pleurotus ostreatus*) production. Afr. J. Microbiol. Res..

[12] Labuschagne PM, Eicker A, Aveling TAS, de Meillon S, Smith MF (2000). Influence of wheat cultivars on straw quality and *Pleurotus ostreatus* cultivation. Biores. Technol..

[13] Fideghelli, C. & De Salvador, F. R. World hazelnut situation and perspectives. In* VII International Congress on Hazelnut* 39–52. 10.17660/ActaHortic.2009.845.2 (2008).

[14] Puliga F, Leonardi P, Minutella F, Zambonelli A, Francioso O (2022). Valorization of hazelnut shells as growing substrate for edible and medicinal mushrooms. Horticulturae.

[15] Cimen F (2007). Characterization of humic materials extracted from hazelnut husk and hazelnut husk amended soils. Biodegradation.

[16] Sürek, E. Prebiotic oligosaccharide production from hazelnut wastes. Doctoral dissertation, (Izmir Institute of Technology, Turkey, 2017).

[17] Guney MS (2013). Utilization of hazelnut husk as biomass. Sustain. Energy Technol. Assess..

[18] Bak T, Karadeniz T (2021). Effects of branch number on quality traits and uield properties of European hazelnut (*Corylus avellana* L.). Agriculture.

[19] Alkaya, E., Altay, T., Ata, A., Çakar, S. O. & Durtas, P. Tarımsal atıklardan yüksek katma değerli biyoürün üretimi. *Ileri teknoloji projeleri destek programı raporu* 35 (2010).

[20] Surek E, Buyukkileci AO (2017). Production of xylooligosaccharides by autohydrolysis of hazelnut (*Corylus avellana* L.) shell. Carbohydr. Polym..

[21] Carrasco-Cabrera CP, Bell TL, Kertesz MA (2019). Caffeine metabolism during cultivation of oyster mushroom (*Pleurotus ostreatus*) with spent coffee grounds. Appl. Microbiol. Biotechnol..

[22] Chai WY, Krishnan UG, Sabaratnam V, Tan JBL (2021). Assessment of coffee waste in formulation of substrate for oyster mushrooms Pleurotus pulmonarius and Pleurotus floridanus. Future Foods.

[23] Peksen A, Yakupoglu G (2008). Tea waste as a supplement for the cultivation of Ganoderma lucidum. World J. Microbiol. Biotechnol..

[24] Kacar, B. Chemical analysis of plant and soil: III, Soil Analysis. Ankara Univ. Faculty of Agriculture, Education Res. Extension Found, Ankara (1994).

[25] TAPPI T 204 CM-17 Solvent Extractives of Wood and Pulp, Standard by Technical Association of the Pulp and Paper Industry (2017).

[26] Wise LE, John EC (1952). Wood chemistry.

[27] TAPPI T203 cm-09 Alpha-, beta- and gamma-cellulose in pulp. TAPPI Press, Atlanta (2009).

[28] Pekşen A, Küçükomuzlu B (2004). Yield potential and quality of some *Pleurotus* species grown in substrates containing hazelnut husk. Pak. J. Biol. Sci..

[29] Fanadzo M, Zireva DT, Dube E, Mashingaidze AB (2010). Evaluation of various substrates and supplements for biological efficiency of *Pleurotus sajor-caju* and *Pleurotus ostreatus*. Afr. J. Biotech..

[30] Atila F (2019). Compositional changes in lignocellulosic content of some agro-wastes during the production cycle of shiitake mushroom. Sci. Hortic..

[31] Membrillo I, Sanchez C, Meneses M, Favela E, Loera O (2011). Particle geometry affects differentially substrate composition and enzyme profiles by *Pleurotus ostreatus* growing on sugar cane bagasse. Biores. Technol..

[32] Ananbeh KM, Almomany AR (2005). Production of oyster mushroom *Pleurotus ostreatus* on olive cake agro waste. Dirasat Agric. Sci..

[33] Gençer A, Özgül U (2016). Utilization of common hazelnut (*Corylus avellana* L.) prunings for pulp production. Drvna industrija.

[34] Atila F (2017). Biodegredation of different agro-endustrial wastes through the cultivation of *Pleurotus ostreatus* (Jacq. ex. Fr) Kummer. J. Biol. Environ. Sci..

[35] Jonathan SG, Akinfemi A, Adenipekun CO (2010). Biodegradation and in-vitro digestibility of maize husk treated with edible fungi (*Pleurotus tuber-regium* and *Lentinus subnudus*) from Nigeria. Electron. J. Environ. Agric. Food Chem..

[36] Kuforiji OO, Fasidi IO (2008). Enzyme activities of Pleurotustuber-regium (Fries) singer, cultivated on selected agricultural wastes. Biores. Technol..

[37] Hatakka A (1994). Lignin-modifying enzymes from selected white-rot fungi: Production and role in lignin degradation. FEMS Microbiol. Rev..

[38] Jefferies T (1990). Biodegradation of liqnin-carbohydrate complex. Biodegradation.

[39] Clarke AJ (1996). Biodegradation of Cellulose: Enzymology and Biotechnology.

[40] Mercy B, Sylvester KT, Nathaniel OB (2011). Effects of lignocellulosic in wood used as substrate on the quality and yield of mushrooms. Food Nutr. Sci..

[41] Zhang R, Li X, Fadel JG (2002). Oyster mushroom cultivation with rice and wheat straw. Biores. Technol..

[42] Pujol D (2013). The chemical composition of exhausted coffee waste. Ind. Crops Prod..

[43] Craig AP, Franca AS, Oliveira LS, Irudayaraj J, Ileleji K (2015). Fourier transform infrared spectroscopy and near infrared spectroscopy for the quantification of defects in roasted coffees. Talanta.

[44] Craig AP, Franca AS, Oliveira LS (2012). Evaluation of the potential of FTIR and chemometrics for separation between defective and non-defective coffees. Food Chem..

[45] Munyendo L, Njoroge D, Hitzmann B (2021). The potential of spectroscopic techniques in coffee analysis—A review. Processes.

[46] Liu R, Huang Y (2005). Structure and morphology of cellulose in wheat straw. Cellulose.

[47] Bari E (2015). Comparison between degradation capabilities of the white rot fungi *Pleurotus ostreatus* and Trametes versicolor in beech wood. Int. Biodeterior. Biodegradation.

[48] Yan F, Tian S, Du K, Wang X (2021). Effects of steam explosion pretreatment on the extraction of xylooligosaccharide from rice husk. BioResources.

[49] Razavi Z, Mirghaffari N, Rezaei B (2015). Performance comparison of raw and thermal modified rice husk for decontamination of oil polluted water. Clean: Soil, Air, Water.

[50] Adapa PK, Tabil LG, Schoenau GJ, Canam T, Dumonceaux T (2011). Quantitative analysis of lignocellulosic components of non-treated and steam exploded barley, canola, oat and wheat straw using fourier transform infrared spectroscopy. J. Agric. Sci. Technol. B.

[51] Craig AP, Botelho BG, Oliveira LS, Franca AS (2018). Mid infrared spectroscopy and chemometrics as tools for the classification of roasted coffees by cup quality. Food Chem..

[52] Lun, L. W., Gunny, A. A. N., Kasim, F. H. & Arbain, D. Fourier transform infrared spectroscopy (FTIR) analysis of paddy straw pulp treated using deep eutectic solvent. In *Advanced Materials Engineering and Technology V AIP Conf. Proc.* (Vol. **1835**)**,** 020049-1-020049-4. 10.1063/1.4981871 (2017).

[53] Akcay C, Yalcin M (2021). Morphological and chemical analysis of Hylotrupes bajulus (old house borer) larvae-damaged wood and its FTIR characterization. Cellulose.

[54] Galletti AMR (2016). Midinfrared FT-IR as a tool for monitoring herbaceous biomass composition and its conversion to furfural. Hindawi Publ. Corp. J. Spectrosc..

[55] Belchior V, Botelho BG, Casal S, Oliveira LS, Franca AS (2019). FTIR and Chemometrics as effective tools in predicting the quality of specialty coffees. Food Anal. Methods.

[56] Baeva E (2020). Evaluation of the cultivated mushroom *Pleurotus ostreatus* basidiocarps using vibration spectroscopy and chemometrics. Appl. Sci..

[57] Swantomo D, Rochmadi R, Basuki KT, Sudiyo R (2013). Synthesis and characterization of graft copolymer rice straw cellulose-acrylamide hydrogels using gamma irradiation. Atom Indonesia.

[58] Reis N, Botelho BG, Franca AS, Oliveira LS (2017). Simultaneous detection of multiple adulterants in ground roasted coffee by ATR-FTIR spectroscopy and data Fusion. Food Anal. Methods.

